# Use of Technology-Based Tools to Support Adolescents and Young Adults With Chronic Disease: Systematic Review and Meta-Analysis

**DOI:** 10.2196/12042

**Published:** 2019-07-18

**Authors:** Jac Kee Low, Elizabeth Manias

**Affiliations:** 1 School of Nursing and Midwifery Centre for Quality and Patient Safety Research Deakin University Burwood Australia

**Keywords:** young adult, adolescent, self-management, transition to adult care, disease management, systematic review

## Abstract

**Background:**

With the large amount of material that is readily available on the internet, there are endless opportunities for electronic health–literate patients to obtain and learn new information. Although novel, a Web- or mobile-based program can be a powerful way to engage adolescents and young adults (AYAs). The ongoing engagement of AYAs with chronic disease is vital not only to empower them but also to ensure a smooth transition from pediatric to adult health care.

**Objective:**

This study aimed to evaluate the current evidence on Web- or mobile-based interventions designed for AYAs.

**Methods:**

This review was registered with PROSPERO: CRD42018096487. A systematic search of MEDLINE Complete, EMBASE, and CINAHL Complete was conducted on April 10, 2019, for studies that examined the perspectives of transition-age patients about technology-based interventions, the process involved in intervention development, or the evaluation of intervention efficacy. For each study, the comprehensiveness of reporting was appraised. The Downs and Black checklist was used for intervention efficacy trials, the Standards for Reporting Qualitative Research checklist was used for qualitative work, and a 16-item tool developed by Tong et al was used for questionnaire research.

**Results:**

The search uncovered 29 relevant studies, which included qualitative studies (n=14), intervention efficacy studies (n=7), questionnaire studies (n=4), mixed qualitative and questionnaire studies (n=2), and a mixed qualitative and pilot randomized controlled trial study (n=1). The reporting comprehensiveness score of questionnaires was rated considerably lower (n=6, 13%-57% [2/16-8/14]) than the scores of intervention efficacy trials (n=8, 48%-85% [13/27-23/27]) and qualitative research (n=17, 40%-93% [8.5/21-19.5/21]). AYAs were receptive to obtaining information via a website or mobile app. An intervention was more likely to be perceived as useful by AYAs when there was a concerted effort to involve AYAs and subject matter experts in the process of intervention design, as opposed to relying solely on the AYAs or the experts alone. The preferred medium of intervention delivery varied greatly for AYAs, ranging from static text to audiovisual materials. However, AYAs considered being concise was the most important aspect. Across different conditions, AYAs were interested in receiving information on diverse topics, such as anxiety and stress management, dealing with insurance, and having social relationships. Patients also requested for disease-specific information, such as weather forecasts and pollen levels for patients with asthma and information related to the pretransplant period for organ transplant recipients. Meta-analyses showed no significant group differences across time on quality of life, self-efficacy, and self-management.

**Conclusions:**

Owing to the lack of intervention efficacy trials, no conclusion can be drawn if an intervention delivered via a mobile app is better than that delivered via a website. However, through this systematic review, it is confirmed that AYAs were receptive to receiving medical information electronically.

## Introduction

### Background

The number of children living with a chronic disease has grown over the past few decades [1-4]. For example, the incidence rate of childhood type 1 diabetes continues to rise by approximately 3% per annum [1,2], whereas the prevalence of cancer increases at 0.6% per annum [4]. The increase in incidence and prevalence may be attributable to medical advances that improve screening and diagnosis in addition to disease management, which altogether offer a better chance of patient survival. One such example is sickle cell disease, which had very few children surviving into adulthood in the 1970s, but 95% of those born in the recent decade will reach their 18th birthday [5]. The change in health care need is inevitable as medical advances are made. To receive age-appropriate care, young patients with pediatric-onset chronic disease need to transition from pediatric to adult health care. Transition is defined as the planned process of preparing adolescents and young adults (AYAs) as they move from caregiver-directed care in a pediatric unit to disease self-management in an adult unit [6].

Although AYAs are no longer children, they are yet to identify themselves as adults. They may be reluctant to detach from the pediatric unit, they may not feel comfortable in the adult care environment, and they may be fearful of their own future after confronting older and sicker patients in the adult unit [7]. If they are not prepared adequately, the transfer to an adult care environment can be problematic, which could cause clinic nonattendance and treatment nonadherence [8-14]. Evidence shows that outpatient clinic attendance among AYAs with chronic diseases, such as type 1 diabetes and sickle cell disease, declines significantly when comparing the pretransfer period with the posttransfer period [8-10]. A study conducted in the United Kingdom reported that 98% of 229 young people with diabetes attended a clinic appointment at least 6-monthly 2 years pretransfer but the proportion declined to 61% at 2 years posttransfer [11]. In a 2015 systematic review, Heery et al [12] reported that between 28% and 63% of adults with congenital heart disease had ≥2 years lapse in care after leaving the pediatric care in Canada, the United Kingdom, and the United States. In a retrospective study involving liver transplant recipients, immunosuppressive medication adherence significantly decreased over time from pretransfer to 2 years posttransfer [14]. These are worrying trends. Not only does patient nonadherence exacerbate symptoms and cause disease progression, but it also leads to the eventual need for more intensive monitoring and expensive treatment. However, there is evidence that an age-appropriate transition program can improve patient outcomes, which include attendance rates and medication adherence in adolescents with inflammatory bowel disease [13] and disease-specific knowledge and satisfaction with care in those with juvenile arthritis [15]. Hence, how the process of transition is managed plays a crucial role in the ongoing engagement of AYAs with the health care system.

To facilitate a successful transition, AYAs with chronic disease need to be equipped with self-management skills and be engaged with their treatment plan to maintain positive health outcomes [16]. A Cochrane review by Campbell et al [17], which was conducted in 2016, only found 4 small randomized controlled trials with sample sizes that ranged from 26 to 81. The trials covered a limited range of interventions, which included a 2-day face-to-face–delivered workshop [18], an 8-month Web-based and text-delivered disease management and skill-based intervention [19], a one-off meeting with a nurse [20], and a structured transition program involving a transition coordinator over a 12-month period [21]. No firm conclusions could be drawn from the intervention studies [17].

### Objective

An emerging area that is worth exploring is the use of Web- or mobile-based materials to engage AYAs. It can be an innovative way to build their skills and prepare them for the transition process [22,23]. As parental and clinician assumptions may fall short of identifying the needs of AYA patients, it is critical to obtain knowledge of AYAs’ perspectives. This knowledge will help ensure that the needs of AYA patients are addressed and that AYAs are appropriately supported through the designed intervention during their transition from pediatric to adult health care. This systematic review aimed to evaluate the current evidence on Web- or mobile-based interventions by summarizing studies that examined either the perspectives of AYAs or intervention efficacy.

## Methods

### Protocol and Registration

The protocol for this systematic review was registered with PROSPERO 2018: CRD42018096487 [24]. It was conducted in accordance with the Preferred Reporting Items for Systematic Reviews and Meta-Analyses guidelines (PRISMA) [25].

### Eligibility Criteria

Studies that met the following criteria were included in the review:

- There were no restrictions on the study design, provided that it was primary research exploring the perspectives of patients about a technology-based intervention; a methods paper describing the intervention development process; or primary research evaluating intervention effectiveness.
- The intervention must be freely available through a device that can be connected to the internet in the form of an app on a mobile device or on the World Wide Web; designed for patients with at least one chronic disease; and accessible by patients at any time.

- Study participants could be aged less than 18 years or adults (aged ≥18 years) who were either transitioning or had already transitioned to adult health care services.

Studies were excluded based on the following criteria:

- The only aim of the technology component of the intervention was to allow participants to engage with another party online, such as forums and social media platforms; test a serious game; test an equipment, such as a Bluetooth spirometer and blood glucose monitors; and remotely monitor patient progress, such as patient portals and symptom reporting platform.
- Studies where pediatric group findings could not be delineated from research involving other patient groups, such as middle-aged persons and older patients.

### Search Strategy

Overall, 3 electronic databases were searched: MEDLINE Complete via EBSCOhost (1967 to March 31, 2019), EMBASE (1972 to March 31, 2019), and CINAHL Complete via EBSCOhost (1978 to March 31, 2019). The search utilized terms associated with the concepts of *technology*, *transition* or *disease management*, *chronic disease*, and *adolescents* or *young adults*. An example of the search strategy is included in Multimedia Appendix 1.

### Study Selection

Search results were collated in a reference manager (EndNote X8, Clarivate Analytics, 2017), duplicates were deleted, and the results were exported to a spreadsheet (Microsoft Excel, Microsoft Corporation, 2016) for initial screening of titles and abstracts. The screening was conducted independently by 2 reviewers (JKL and EM) whereby a priori inclusion and exclusion criteria were applied. The same reviewers further reviewed the full texts of articles independently to select studies for inclusion according to the eligibility criteria. Manual checks on the reference lists of retrieved reviews on the relevant topic were conducted to identify articles not found by the database searches. Discordance between reviewers was resolved through discussion.

### Data Extraction and Quality Assessment

For each included study, the following items were extracted: study characteristics, participant demographics, and study design using a standardized form entered into Microsoft Excel. Data related to the design of a technology-based intervention were also extracted.

For studies using multiple methods, the comprehensiveness of reporting was appraised using checklists applicable to the major methodological approach of each study. The Downs and Black checklist (D&B) was used for intervention efficacy trials [26], the Standards for Reporting Qualitative Research (SRQR) checklist was used for qualitative research [27], and a 16-item tool developed by Tong et al [28] was used for questionnaire research. Owing to the lack of clarity on how to score item 27 in the D&B checklist (power calculation), a score of 0 or 1 was allocated to indicate whether the authors achieved their target sample size or not. If no power calculation was conducted a priori, a score of 0 was given for item 27. The same approach has been used by previous researchers [29,30]. No study was excluded based on the quality assessment.

### Data Synthesis and Analysis

Data collected from all qualitative studies, including mixed-methods, exploratory, and feasibility studies, using interviews and focus groups in addition to open-ended responses to items in questionnaires, were extracted and organized using NVivo (version 11, QSR International Pty Ltd). Qualitative data were pooled and thematically analyzed using the method outlined by Thomas and Harden [31] by a reviewer (JKL). Quantitative data were pooled for meta-analyses using RevMan (version 5.3, The Cochrane Collaboration). Where a meta-analysis could not be conducted as a result of the small number of available studies, a descriptive synthesis of the quantitative findings was undertaken instead.

## Results

### Study Selection

**Figure 1:**
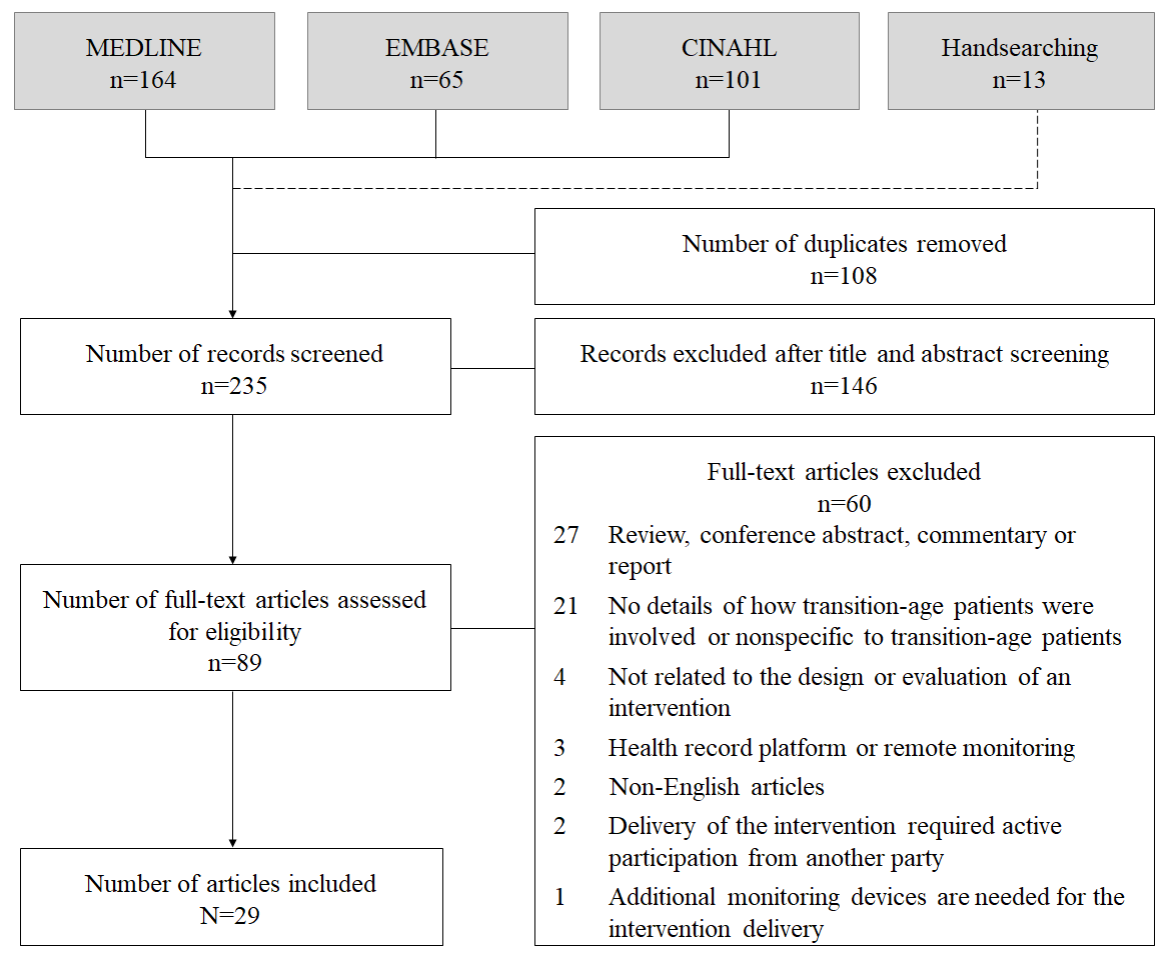
Study flow diagram.

The initial search yielded 235 records. Of these, 89 were selected for full-text review, which led to further exclusion of 60 studies because of ineligibility (Figure 1). Overall, 29 studies were included in the review [19,32-59].

### Study Characteristics (N=29)

All studies (N=29) were conducted in developed countries (Table 1). Diabetes was the most investigated type of chronic disease (n=9) followed by rheumatic disease (n=6), asthma (n=5), and cystic fibrosis (n=4), whereas 2 studies did not specify the type of chronic disease examined [32,35]. Participants were aged between 7 and 28 years (Table 2).

A total of 17 studies utilized a qualitative approach: 6 exploratory studies [32,39,51,53,54,57], 3 multiphase studies involving the iterative design and development of an intervention [43,47,52], 2 feasibility studies [34,45], 2 usability studies of an intervention [42,55], 1 evaluation of user experience [50], and 3 mixed-methods studies [35,38,58].

Among the included studies, 6 studies had a questionnaire component in the study design: a feasibility study [33], a cross-sectional survey to inform the development of an eHealth intervention [40], a multiphase study involving the iterative development and user evaluation of an intervention [36], an evaluation of user engagement [59], and 2 mixed-methods studies [35,38].

In addition, 8 studies included an intervention efficacy trial, of which one was a mixed-methods study [58]. The conduct of a randomized controlled trial was the most popular design (n=3) [19,41,44] followed by a pilot randomized controlled trial (n=3) [37,56,58]. The remaining intervention efficacy trials utilized a nonrandomized controlled study design [48] and a pretest -posttest design [46].

**Table 1:** Description of included studies (N=29).

Study identity	Country	Study design	Type of chronic disease	Quality assessment
Abraham et al [32]	United States	Exploratory qualitative design using semistructured interview	—^a^	SRQR^b^: 15.5/21
Ammerlaan et al [33]	Netherlands	Quantitative feasibility study using a Web-based questionnaire	Rheumatic disease	Tong^c^: 7/13
Ammerlaan et al [34]	Netherlands	Qualitative feasibility study using semistructured interview	Juvenile idiopathic arthritis	SRQR: 17/21
Applebaum et al [35]	United States	Mixed methodologies, cross-sectional study: questionnaire and qualitative using focus group	—	Tong: 8/14; SRQR: 9/21
Ashurst et al [36]	United Kingdom	2-stage approach: stage 1 development and stage 2 evaluation using email and a Web-based questionnaire	Diabetes	Stage 2 Tong: 6/16
Breakey et al [37]	Canada	Pilot randomized control trial	Hemophilia	D&B^d^: 19/27
Coyne et al [38]	Ireland	Four-phase participatory iterative approach using questionnaire, one-to-one interview, participatory workshop, and Google Analytics	Diabetes, cystic fibrosis, or congenital heart disease	Phase 1 Tong: 2/14; Phase 1 SRQR: 11/21
Huang et al [39]	United States	Exploratory qualitative design using a focus group	Diabetes, cystic fibrosis, or IBD^e^	SRQR: 15/21
Huang et al [19]	United States	Randomized controlled trial	Diabetes, cystic fibrosis, or IBD	D&B: 23/27
Johnson et al [40]	United States	Cross-sectional study: Web-based questionnaire	Juvenile arthritis	Tong: 7/14
Joseph et al [41]	United States	Randomized controlled trial	Asthma symptoms	D&B: 19/27
Korus et al [42]	Canada	Qualitative usability testing approach using semistructured interview	Solid organ transplant recipient	SRQR: 15/21
Lopez et al [43]	United States	Formative iterative process using semistructured interview and group interview	Congenital heart disease	SRQR: 17/21
Mulvaney et al [44]	United States	Randomized controlled trial	Diabetes	D&B: 15/27
Mulvaney et al [45]	United States	Qualitative feasibility study	Diabetes	SRQR: 11.5/21
Paul [46]	Australia	Two-phase, multimethod approach: phase 1 evaluation of intervention fidelity and phase 2 feasibility pre- to posttest	Diabetes	Phase 2 D&B: 14/27
Peters et al [47]	Australia	Multiphase, participatory user research study using participatory workshop, workbook, and user evaluation	Asthma	SRQR: 16/21
Runge et al [48]	Germany	Nonrandomized trial	Asthma	D&B: 17/27
Scal et al [49], Secor-Turner et al [60]	United States	Descriptive paper on the intervention development process	Juvenile arthritis	N/A^f^
Schneider et al [50]	United States	Qualitative evaluation of user experience using semistructured interview	Asthma	SRQR: 14/21
Schneider et al [51]	United States	Exploratory qualitative design using semistructured interview	Asthma	SRQR: 18/21
Simmons et al [52]	United States	Multiphase, iterative design and development of an intervention: environmental scan, Web-based or telephone focus group, and in-person focus group	Hemophilia	SRQR: 13.5/21
Slater et al [53]	Australia	Exploratory qualitative design using semistructured interview and focus group	Musculoskeletal pain	SRQR: 19/21
Sterling et al [54]	Canada	Exploratory qualitative design using semistructured interview	Hemophilia	SRQR: 15/21
Stinson et al [55]	Canada	Qualitative usability testing with semistructured interview	Juvenile idiopathic arthritis	SRQR: 18.5/21
Stinson et al [56]	Canada	Pilot randomized controlled trial	Juvenile idiopathic arthritis	D&B: 21/27
Stinson et al [57]	Canada	Descriptive exploratory qualitative design using focus group and interview	Chronic pain	SRQR: 19.5/21
Whittemore et al [58]	United States	Multiphase mixed-methods design: qualitative using focus group and think-aloud method, followed by a feasibility and pilot study	Diabetes	Phase 1 SRQR: 8.5/21; phase 2 D&B: 13/27
Zhao et al [59]	Australia	Pilot study using questionnaire	Diabetes, cystic fibrosis, or IBD	Tong: 2/16

^a^Not specified or not reported.

^b^SRQR: Standards for Reporting Qualitative Research.

^c^Tong: the 16-item checklist questionnaire developed by Tong et al [28].

^d^D&B: Downs and Black checklist for intervention efficacy trial.

^e^IBD: inflammatory bowel disease.

^f^N/A: not applicable.

**Table 2:** Description of participant demographics.

Study identity	Number of participants^a^	Mean age (SD or range)^a^	Age group (n)^a^	Gender (male), n (%)^a^
Abraham et al [32]	20	—^b^	7-11 (4), 12-14 (9), 15-17 (7)	8 (40)
Ammerlaan et al [33]	IG^c^: 10; CG^d^: 9	IG: 22.3 (17-25); CG: 20.7 (17-25)	—	IG: 1 (10); CG: (2) 22
Ammerlaan et al [34]	13	20 (17-22)	—	1 (8)
Applebaum et al [35]	Questionnaire: 35; Focus group: 20	Questionnaire: 16.9 (13-20); Focus group: —	—	Survey: 9 (26); Focus group: —
Ashurst et al [36]	Stage 1: 6; Stage 2: 83	Stage 1: 20.3 (3.3); Stage 2: 19.0 (2.6)	—	Stage 1: —; Stage 2: 37 (45)
Breakey et al [37]	29	IG: 16.0 (1.4); CG: 16.1 (1.4)	—	29 (100)
Coyne et al [38]	Phase 1 questionnaire: 207, interview: 21; phase 2 co-design group: 5, telephone interview: 4, participatory workshop: 12	—	Phase 1 questionnaire: 14-25 (207), interview participants: 14-25 (21); phase 2 co-design group: 15-25 (5), telephone interview: 15-25 (4), participatory workshop: 15-25 (12)	Phase 1 questionnaire: —, interview participants: —; phase 2 co-design group: 2 (40), telephone interview: —, participatory workshop: —
Huang et al [39]	10	20 (18-25)	—	4 (40)
Huang et al [19]	IG: 40; CG: 41	IG: 17 (12-20)^e^; CG: 17 (12-19)^e^	—	IG: 17 (43); CG: 20 (49)
Johnson et al [40]	134	High PedsQL_Psycho: 15.9 (—); low PedsQL_Psycho: 16.3 (—)	—	High PedsQL_Psycho: 10 (15); low PedsQL_Psycho: 12 (18)
Joseph et al [41]	314	15.3 (1.0)	—	115 (37)
Korus et al [42]	21	—	12-14 (7), 15-17 (13), 18 (1)	14 (67)
Lopez et al [43]	Phase 2 expert panel: 6	Phase 2 expert panel: 16 (15-19)^e^	—	Phase 2 expert panel: 2 (33)
Mulvaney et al [44]	IG: 48; CG: 24	IG: 15.1 (1.5); CG: 15.1 (1.3)	—	IG: 25 (52); CG: 15 (62)
Mulvaney et al [45]	41	IG only: 15.1 (1.5)	—	IG only: 21 (51)
Paul [46]	Phase 1: —; phase 2: 5 (*Only 3 participants completed phase 2)*	—	Phase 1: —; phase 2: 14 (1), 15 (3), 17 (1)	Phase 1: —; phase 2: 3 (60)
Peters et al [47]	20	17.8 (15-24)	—	8 (40)
Runge et al [48]	178	IG1: 11.1 (2.4); IG2: 11.0 (2.2); CG: 11.5 (2.9)	—	IG1: 47 (55); IG2: 29 (66); CG: 38 (79)
Scal et al [49], Secor-Turner et al [60]	Youth: 5; Young adults: 5	Youth:16.2 (14-21)^f^; Young adults: 25.4 (22-28)^f^	—	Youth: 2 (40)^f^; young adults: 1 (20)^f^
Schneider et al [50]	16	—	13-18 (16)	—
Schneider et al [51]	20	14.4 (1.6)	—	9 (45)
Simmons et al [52]	Web-based focus group: 40; In-person message testing focus group: 19	—	Web-based focus group: 16-17 (24), 18-19 years (16); In-person message testing focus group: 16-17 (12), 18-19 (7)	—
Slater et al [53]	23	20.8 (2.4)	—	3 (13)
Sterling et al [54]	11	16.3 (12.8-18.3)	—	11 (100)
Stinson et al [55]	19	15.7 (1.5)	—	5 (26)
Stinson et al [56]	IG: 22; CG: 24	IG: 14.4 (1.3); CG: 14.8 (1.7)	—	IG: 7 (32); CG: 8 (33)
Stinson et al [57]	23	—	14-18 (23)	5 (22)
Whittemore et al [58]	Phase 1 intervention development: 3; Phase 2 randomized pilot trial: 12; program evaluation: 10	Phase 1 intervention development: —; phase 2 randomized pilot trial: 14.4 (0.9); program evaluation: 14.0 (1.2)	—	Phase 1 intervention development: —; phase 2 randomized pilot trial: 5 (42); Program evaluation: 6 (60)
Zhao et al [59]	10	20.2 (—)	—	3 (30)

^a^Characteristics of parent, health care professional, or healthy participants are not included.

^b^Not specified or not reported.

^c^IG: intervention group.

^d^CG: comparison group.

^e^Median years (minimum-maximum).

^f^Information was obtained from a related, secondary source.

### Comprehensiveness of Reporting

An evaluation of the comprehensiveness of reporting was conducted for all studies, except for Scal et al [49] as the authors did not present any qualitative or quantitative data, and it merely provided a description of the intervention development process. All mixed-methods studies were assessed using 2 separate checklists [35,38,58]. One study, which was identified by the authors as a mixed-methods approach, was actually a study involving multiple methods including a literature review, Web-based or telephone focus group, and in-person focus group [52]. Overall, the reporting of questionnaires was rated considerably lower (n=6, scores from the questionnaire developed by Tong et al [28]: 13%-57% [2/16-8/14]) than the intervention efficacy trials (n=8, D&B scores: 48%-85% [13/27-23/27]) and qualitative research (n=17, SRQR scores: 40% -93% [8.5/21-19.5/21]).

Of the 17 qualitative research papers, 4 were missing at least 40% of the items considered important on the SRQR checklist (Multimedia Appendix 2). Most studies were lacking in detail on the data processing of participants’ responses before data analysis, such as the procedure to ensure anonymity and rigor (n=14), an indicative title containing information that it was a qualitative study (n=13), the characteristics of the data collector that may influence the research such as qualification and relationship with participants (n=12), and the context from where participants were recruited (n=12).

All 6 studies that included a questionnaire were missing at least 40% of the items considered important on the Tong et al [28] 16-item checklist for reporting questionnaire studies (Multimedia Appendix 3). None of the 6 studies included details on participants’ response rate, characteristics of refusals, if follow-up reminder was provided, and whether the questionnaire was piloted.

Of the 8 studies that reported an intervention efficacy trial, 3 were missing at least 40% of the items considered important on the D&B checklist (Multimedia Appendix 4). All the studies did not detail how the sample size was estimated, a majority did not detail if blinding of participants and data collectors occurred (n=7) and if there were any adverse events as a result of the intervention or the lack of it (n=7). In 5 studies, the reporting of the results was not ideal [19,37,41,44,58]. For example, 3 studies reported within-group comparisons, whereas the reporting of between-group comparisons across time was lacking in detail [19,37,44]. One study reported having the intervention group showing trends for “better diabetes self-efficacy, better general treatment and less perceived stress” [58, p.7] than the control group; however, the reported *P* values were .20, thereby, demonstrating a lack of statistical significance.

### Summary of Interventions (n=22)

Of the 22 studies which provided a description of the intervention (Multimedia Appendix 5), 8 were evaluated in an efficacy trial. All of the 8 evaluations were conducted on Web-based interventions [19,37,41,44,48,56,58].

Most interventions were delivered via a website (n=13) [19,34,37,38,41,42,44,46,48,49,52,55,56,58]. Other modes of delivery included a mobile app (n=4) [47,50,57,59] and a choice of 3 websites and 3 mobile apps developed by peers (n=1) [36]. In addition, 8 interventions were based upon theories, such as Social Cognitive Theory, Self-Determination Theory, Self-Efficacy Theory, and Social Learning Theory [19,33,44,46,47,49,57,58].

### Adolescents and Young Adults’ Perceived Usefulness or Acceptability (n=16)

Perceived usefulness or acceptability was explored in 14 interventions across 16 studies because 2 of the interventions, *Challenge your arthritis* [33,34] and *Teens Taking Charge: Managing Arthritis Online* [55,56] were evaluated twice (Multimedia Appendix 6). Most of the interventions (n=11) received a positive reaction from the participants [33,34,37,42,44,46,47,51,52,55-58]. Of the 3 interventions that received a neutral response, 2 did not involve pediatric patients in the intervention development [50,59], whereas one fully relied on AYAs without the involvement of a health care professional or content expert [36].

### Adolescents and Young Adults’ Preferred Intervention Design

The use of preexisting technology, such as a mobile app, not designed specifically for AYAs was not appealing to them [50]. As indicated by 91% of AYAs in the study conducted by Ashurst et al [36], AYAs’ input was important to ensure that the intervention was tailored to their needs.

#### Preferred Delivery Method

AYAs’ preferred delivery method ranged from concise text to interactive content [50,53,58]. Placement of the information was important, whereby one study reported that AYAs wanted the most important information on each page at the top of the page [55]. Adolescents suggested incorporating visually appealing features, such as pictures and graphics [55], and games or audiovisual medium to create an engaging website [42,43,51,54]. Learning about medications through interactive games on a tablet or watching educational videos at a kiosk was also preferred [32]. Quizzes were considered more engaging than the sole use of text [54]. In addition, visual aids that allowed patients to view the severity level of their condition based on their symptoms were found be useful [50].

The main advantage of a Web- or mobile-based intervention was that it was readily accessible and it could be browsed at any time at the AYA’s preferred pace [38,39]. To optimize uptake, AYAs suggested that a mobile app should be affordable and be made available on major mobile platforms whilst a website should be mobile-optimized [53]. Furthermore, an app that could perform offline would further optimize usage [51].

Short videos were very appealing to AYAs, especially those that provide support on the medical, lifestyle, and psychological aspects of living with a chronic disease [50,52]. Video testimonial by young people was deemed as an important way for AYAs to realize that they were not alone in their struggles [38]. In those interventions that incorporated videos or life stories, AYAs were able to understand the content easily and enjoyed watching the videos because they recognized themselves in the stories relayed [34,42,52].

#### Information Needs

AYAs suggested using peers with chronic disease to comment on topics such as disease management tips, transition experiences [39], and disease experiences [54]. In addition, AYAs would like to receive disease-specific news or research updates [39,54]. In preparation for their transition to adult care, AYAs sought to obtain practical information, such as the differences between child and adult health care, the clinic’s location, and key staff members [38].

Across different conditions, mental health support was found to be an appreciated feature by AYAs [34,39,47]. Huang et al [39] reported that young adults wanted to learn how to manage anxiety and stress, intimate relationships, alcohol and drug situations, and health insurance regardless of their condition. Similarly, Ammerlaan et al [34] reported that the most appealing topics were on how to deal with pain, fatigue and emotions, physical exercise, holidays, study, and work. Hence, rather than emphasizing health care and medical information, AYAs preferred to learn about challenges at school, work, and social settings and the emotional burden that they would be experiencing [49].

Suggestions to include disease-specific information were mentioned by AYAs. For example, patients with asthma mentioned about incorporating real-time reports concerning environmental conditions, such as pollen levels and weather forecasts, within an app [50]. They wanted a mobile app that could be customized to include their own profile with a personal medical history and treatment summary, reminders, a symptom tracker, access to emergency information even in the absence of an internet connection, and motivational feedback [47,51]. Conversely, young adults with juvenile idiopathic arthritis wanted reliable sources of medication information to be accessible via the website [34]. For organ transplant recipients, they wanted to have information relevant to the pretransplant period, details about medical emergencies or complications, and a section for parents [42]. Patients with hemophilia sought to learn about the pathology and severity of hemophilia, first aid, emergency services, and activity limitations [54].

#### Support From Peers or Health Care Professionals

Compared with face-to-face interactions, AYAs preferred an online support group [40,54]. One study reported that AYAs were wary of using chat rooms and existing social media sites to talk to strangers because of privacy concerns [35]. However, 7 other studies reported that AYAs would like to have the opportunity to network with their peers [34,39,42,43,53-55], which could help to ease feelings of isolation. Online discussion forums [34,42,54] or *question and answer* forums or use of a platform to share achievements [47] were suggested by AYAs as ways that they could feel connected with others diagnosed with the same condition. Although they might not post to the forum, 96% of AYAs revealed that reading comments posted by others was useful [45]. Some AYAs liked being able to contact or share information with their health care providers [35,47,50,51].

### Synthesis of Quantitative Data (n=8)

A total of 8 studies included an intervention efficacy trial, and all evaluations were conducted on Web-based interventions; none included a mobile app (Multimedia Appendix 7). For one study that had 3 groups of participants, the evaluation of intervention efficacy was reported based on the comparison between the “standardized patient management program (SPMP)” group and the “internet-based education program plus standardized patient management program (IEP+SPMP)” group as opposed to the “usual care” group [48]. Meta-analysis did not include studies that had no control group [46] and those that did not report standard deviation [48,58] as group comparisons could not be conducted. Information requests were sent but there was no response from the corresponding authors [48,58].

The most frequently measured outcomes were quality of life, self-efficacy, and self-management. The combined data for meta-analysis showed that there was no statistically significant group difference in quality of life (n=3, standardized mean difference −0.15, 95% CI −0.52 to 0.22; *P*=.43), self-efficacy (n=3, standardized mean difference 0.15, 95% CI −0.17 to 0.47; *P*=.23), and self-management (n=3, standardized mean difference 0.11, 95% CI −0.18 to 0.40; *P=*.44).

## Discussion

### Principal Findings

This systematic review examined 29 articles, which were published between 2006 and 2019, and included primary research articles discussing Web- and mobile-based interventions.

Using the qualitative data, this systematic review revealed that AYAs were very receptive to obtaining information electronically. AYAs were more likely to perceive an intervention as useful when there was a concerted effort of involving AYAs and experts in the process of intervention design as opposed to relying solely on AYAs or experts alone. However, engaging AYAs in research could be difficult. Ashurst et al [36] reported that many AYAs cited study commitment as a reason for nonparticipation or withdrawal although the project was conducted at a time that coincided with school holidays. Similarly, Simmons et al [52] faced difficulties in recruiting participants although an extensive strategy was used, and a cash incentive was offered. With the growing consensus about the crucial role that patients play in improving the value of health care research, there is a clear need to identify the best methods to achieve engagement, which is currently lacking [61]. To engage AYAs, research suggests that the most effective recruitment approach may be one that is initiated by AYAs’ own health care providers combined with social media outreach and frequent contact [62].

Although AYAs had different preferred styles of message delivery, ranging from static text to audiovisual materials, being concise was the most important part to keeping them engaged. The findings of this systematic review revealed approaches that can be undertaken to design an intervention for AYAs; however, it also contains suggestions suitable for young school-aged children. The use of engaging technology can be a fun and easy way to captivate patients’ attention and to encourage learning [32,35,53]. For example, young school-aged children can benefit from an intervention with audiovisual content about self-management and their condition. However, depending on the age group that the intervention is targeting, there is a need to use age-appropriate language.

Using the combined quantitative data, the meta-analysis showed that efficacy of the interventions on quality of life, self-efficacy, and self-management skills could not be found. With only 3 sets of data available for each outcome, the insignificant findings could be because of the study heterogeneity (I^2^ score ranged from 27% for self-management to 50% for quality of life). A conclusion cannot be drawn on the overall effect of a Web-based intervention in preparing AYAs to self-manage their condition and to become independent adults in comparison with usual care. As there was no intervention efficacy trial on a mobile app, no conclusion can be drawn if a mobile app is a better tool for AYAs than a website. There was also a lack of high-quality randomized controlled trials as only 3 intervention efficacy trials obtained a D&B score of ≥20 out of a possible 27 items. The omission of information in intervention efficacy reports made it hard to assess if there were biases that could have influenced the findings. In particular, the reports did not detail the characteristics of participants who were lost to follow-up, and if blinding of participant and data collector was achieved. Similarly, studies that utilized a questionnaire failed to detail response rates and if the questionnaire used was piloted before distribution. The methodological shortcomings make it difficult for future investigators to adopt or refine the strategies used in designing or refining an intervention.

To date, systematic reviews have been conducted to examine the use of technology-based interventions in young people, such as the prevention and treatment of pediatric obesity [63] and suicide prevention [64]. Like the findings of this systematic review, other researchers found that there is a paucity of current evidence for technology-based interventions to improve patient outcomes [63,64]. The use of a Web- or mobile-based intervention is a relatively new area. As evidenced in this systematic review, the oldest study retrieved was published in 2006. There is a rise in the number of research studies undertaken given that most studies (n=17, 61%) were published between 2015 and 2019. Although there are many trials exploring AYAs’ perspectives, quality randomized controlled trials assessing the efficacy of an intervention are lacking.

### Limitations

The findings of this systematic review and meta-analysis should be interpreted with caution because of several methodological limitations. First, in the hope of uncovering replicable and inexpensive interventions, this systematic review focused on interventions that did not require extra resources, such as a device or a third party. However, it was found that AYAs wanted to obtain support from their peers or health professionals online. The extent of how this support enhanced the effectiveness and the reach of an intervention could not be concluded from this systematic review. Second, the included studies consisted of qualitative, quantitative, and mixed-methods studies, making it difficult to compare the quality of the studies. The different study designs also meant that the data were so varied that it was difficult to ascertain the effects of the interventions on overall AYAs’ transition readiness. Nonetheless, the findings provided information on AYAs’ preferred intervention design for future work. Finally, although there were no language restrictions when searching the major medical databases, it was acknowledged that a search including foreign language databases may reveal additional studies published in languages other than English in developed and developing countries.

### Conclusions

This systematic review revealed that AYAs were receptive to receiving information through a website or mobile app, which is a first step to engaging them in their own care. Although no conclusion can be drawn on an effective intervention design because of the lack of intervention efficacy trials, this systematic review contained information about AYAs’ preferred intervention. In designing an AYA-focused intervention, the best approach would be to first identify AYAs’ disease-specific needs. This is to be coupled with or followed by obtaining suggestions from health professionals caring for AYAs. Finally, it is essential to obtain AYAs’ feedback on the style and content of the designed intervention. Such a systematic iterative process will ensure that the designed intervention is accepted by AYAs, in the hope that it will improve patient engagement during the transition process and, thus, patient outcomes. Providing AYAs an age-appropriate, reliable condition-specific resource, which can be accessed anywhere, is the very first step in supporting them to becoming resourceful independent adults managing their own care.

## Appendix

Multimedia Appendix 1 Search strategy.

Multimedia Appendix 2 Quality appraisal of the qualitative component of the studies.

Multimedia Appendix 3 Quality appraisal of the questionnaire component of the studies.

Multimedia Appendix 4 Quality appraisal of intervention efficacy trials.

Multimedia Appendix 5 Characteristics of included interventions.

Multimedia Appendix 6 A summary of feasibility study or users’ perceived acceptance of theintervention (n=16).

Multimedia Appendix 7 A summary of intervention efficacy evaluations (n=8).

## Abbreviations

AYAs: adolescents and young adults
D&B: Downs and Black checklist
SRQR: Standards for Reporting Qualitative Research
