# Supplementary material for: Use of Technology-Based Tools to Support Adolescents and Young Adults With Chronic Disease: Systematic Review and Meta-Analysis
Source: JMIR Mhealth Uhealth. 2019 Jul 18;7(7):e12042. doi: 10.2196/12042 (PMC6670279; doi:10.2196/12042)
Supplement: Multimedia Appendix 1 [file mhealth_v7i7e12042_app1.pdf]

Multimedia Appendix 1. Search strategy

Database: EBSCOHost MEDLINE Complete 1967 to 31 March 2019

Search strategy:

1. TI adolescen* OR AB adolescen*
2. TI teen* OR AB teen*
3. TI youth OR AB youth
4. TI "young adult*" OR AB "young adult*"
5. TI "young patient*" OR AB "young patient*"
6. MH adolescent*
7. MH "adult children"
8. MH "young adult"
9. S1 OR S2 OR S3 OR S4 OR S5 OR S6 OR S7 OR S8
10. TI Transition OR AB Transition
11. TI "Patient education" OR AB "Patient education"
12. TI self-management OR AB Self-management
13. TI "Disease management" OR AB "Disease management"
14. TI "Medical management" OR AB "Medical management"
15. (MH "Transitional Care")
16. (MH "Transition to Adult Care")
17. S10 OR S11 OR S12 OR S13 OR S15 OR S16
18. TI Technology OR AB Technology
19. TI “Mobile device” OR AB “Mobile device”
20. TI Internet OR AB Internet
21. TI Web OR AB Web
22. TI Computer OR AB Computer
23. TI "Mobile app*" OR AB "Mobile app*"
24. S18 OR S19 OR S20 OR S21 OR S22 OR S23
25. chronic disease* or chronic illness* or long term condition* or chronic condition*
26. S9 AND S17 AND S24 AND S25
