# Supplementary material for: Use of Technology-Based Tools to Support Adolescents and Young Adults With Chronic Disease: Systematic Review and Meta-Analysis
Source: JMIR Mhealth Uhealth. 2019 Jul 18;7(7):e12042. doi: 10.2196/12042 (PMC6670279; doi:10.2196/12042)
Supplement: Multimedia Appendix 2 [file mhealth_v7i7e12042_app2.pdf]

Appendix 2. Quality appraisal of the qualitative component of the studies

Item	[32]	[34]	[35]	[38]	[39]	[42]	[43]	[45]
**Title and abstract**								
1	No	No	No	No	No	No	Yes	No
2	Yes	Yes	Yes	Yes	Yes	Yes	Yes	Yes
**Introduction**								
3	Yes	Yes	Yes	Yes	Yes	Yes	Yes	Yes
4	Yes	Yes	Yes	Yes	Yes	Yes	Yes	Yes
**Methods**								
5	No	Yes	No	No	No	Yes	No	No
6	No	Yes	No	No	No	No	Yes	No
7	Partially	Partially	Partially	No	Partially	No	Yes	Partially
8	Yes	No	No	No	No	No	Yes	Yes
9	Partially	Partially	Partially	Yes	Partially	Partially	Partially	Yes
10	Yes	Yes	Yes	No	Yes	Yes	Partially	Partially
11	Yes	Yes	Yes	Yes	Partially	Yes	Yes	No
12	Yes	Yes	No	No	Yes	Yes	Yes	Yes
13	Partially	Partially	Partially	No	Partially	Partially	No	No
14	Partially	Yes	Partially	No	Yes	Yes	Yes	Partially
15	Yes	Partially	No	No	Yes	Yes	No	No
**Results/findings**								
16	Yes	Yes	Yes	Yes	Yes	Yes	Yes	Partially
17	Yes	Yes	No	Yes	Yes	Yes	Yes	No
**Discussion**								
18	Yes	Yes	No	Yes	Yes	Yes	Yes	Partially
19	Partially	Yes	Yes	Yes	Yes	Yes	Yes	Yes
**Other**								
20	Yes	Yes	No	Yes	Yes	No	Yes	Yes
21	Yes	Yes	No	Yes	Yes	Yes	Yes	Yes
Score*	15.5	17	9	11	15	15	17	11.5

* Higher score indicates higher quality, with *yes*=1, *partially*=0.5 and *no*=0

Appendix 2: Quality appraisal of the qualitative component of the studies

Item	[47]	[50]	[51]	[52]	[53]	[54]	[55]	[57]	[58]
**Title and abstract**									
1	Yes	No	Yes	No	Yes	No	No	No	No
2	Yes	Yes	Yes	Yes	Yes	Yes	Yes	Yes	Yes
**Introduction**									
3	Yes	Yes	Yes	Yes	Yes	Yes	Yes	Yes	Yes
4	Yes	Yes	Yes	Yes	Yes	Yes	Yes	Yes	Yes
**Methods**									
5	Yes	No	No	No	Yes	No	Yes	Yes	No
6	Partially	No	Partially	No	Yes	No	No	Yes	No
7	Yes	Partially	Yes	Yes	Partially	Partially	Partially	Yes	No
8	No	Yes	Yes	Yes	No	Yes	Yes	Yes	No
9	Partially	Partially	Yes	Yes	Yes	Yes	Yes	Yes	No
10	Yes	Yes	Yes	Yes	Yes	Yes	Yes	Yes	Yes
11	No	Partially	No	Yes	Yes	Yes	Yes	Yes	No
12	Yes	Yes	Yes	Yes	Yes	Yes	Yes	Yes	No
13	No	Partially	Yes	No	Partially	Partially	Yes	Partially	No
14	Yes	Yes	Yes	No	Yes	Yes	Yes	Yes	Yes
15	No	Yes	Yes	Yes	Yes	Yes	Yes	Yes	No
**Results/findings**									
16	Yes	Yes	Yes	No	Yes	Yes	Yes	Yes	Yes
17	Yes	No	Yes	Partially	Yes	Yes	Yes	Yes	Partially
**Discussion**									
18	Yes	Yes	Yes	No	Yes	No	Yes	Yes	Yes
19	Yes	No	Yes	Yes	Yes	Yes	Yes	Yes	No
**Other**									
20	Yes	Yes	Yes	Yes	Yes	No	Yes	Yes	No
21	Yes	Yes	Yes	Yes	Yes	Yes	Yes	Yes	Yes
Score*	16	14	18	13.5	19	15	18.5	19.5	8.5

* Higher score indicates higher quality, with yes = 1; Partially = 0.5; No = 0
