# Supplementary material for: Use of Technology-Based Tools to Support Adolescents and Young Adults With Chronic Disease: Systematic Review and Meta-Analysis
Source: JMIR Mhealth Uhealth. 2019 Jul 18;7(7):e12042. doi: 10.2196/12042 (PMC6670279; doi:10.2196/12042)
Supplement: Multimedia Appendix 3 [file mhealth_v7i7e12042_app3.pdf]

Multimedia Appendix 3. Quality appraisal of the questionnaire component of the studies

Item	[33]	[35]	[36]	[38]	[40]	[59]
**Participant characteristics**						
1	Yes	Yes	Yes	No	Yes	No
2	No	Yes	No	No	No	No
3	No	No	No	No	No	No
4	Yes	Yes	Yes	No	Yes	Yes
5	No	No	No	No	No	No
**Survey administration**						
6	Yes	Yes	Yes	No	Yes	No
7	Yes	Yes	Yes	Yes	Yes	No
8	No	No	Yes	No	Yes	No
9	N/A	No	No	No	No	No
10	No	Yes	No	No	No	No
**Survey design**						
11	No	No	No	No	No	No
12	Yes	No	No	No	No	No
13	Yes	Yes	No	Yes	Yes	No
**Data analysis**						
14	Yes	Yes	No	No	Yes	No
15	N/A	N/A	No	N/A	N/A	Yes
16	N/A	N/A	Yes	N/A	N/A	No
Score*	7	8	6	2	7	2

* Higher score indicates higher quality, with *yes*=1, *no*=0 and *N/A*=not applicable
