# Supplementary material for: Use of Technology-Based Tools to Support Adolescents and Young Adults With Chronic Disease: Systematic Review and Meta-Analysis
Source: JMIR Mhealth Uhealth. 2019 Jul 18;7(7):e12042. doi: 10.2196/12042 (PMC6670279; doi:10.2196/12042)
Supplement: Multimedia Appendix 4 [file mhealth_v7i7e12042_app4.pdf]

Multimedia Appendix 4. Quality appraisal of intervention efficacy trials

Item	[37]	[19]	[41]	[44]	[46]	[48]	[56]	[58]
**Reporting**								
1	Yes	Yes	Yes	Yes	Yes	Yes	Yes	Yes
2	Yes	Yes	Yes	Yes	Yes	Yes	Yes	Yes
3	Yes	Yes	Yes	Yes	Yes	Yes	Yes	No
4	Yes	Yes	Yes	Yes	Yes	Yes	Yes	Yes
5	No	Yes	Yes	No	No	Yes	Yes	No
6	No	No	No	No	Yes	Yes	Yes	No
7	Yes	Yes	No	Yes	No	Yes	No	Yes
8	No	Yes	No	No	No	No	No	No
9	Yes	Yes	Yes	No	No	No	No	No
10	Yes	Yes	Yes	Yes	Yes	Yes	Yes	Yes
**External validity**								
11	Yes	Yes	Yes	Unable to determine	Yes	Yes	Yes	Unable to determine
12	Yes	Yes	Unable to determine	Unable to determine	No	Unable to determine	Yes	Unable to determine
13	Yes	Yes	Yes	Yes	Yes	Yes	Yes	Yes
**Internal validity - bias**								
14	No	No	Unable to determine	Unable to determine	No	No	No	Unable to determine
15	No	Yes	Unable to determine	Unable to determine	No	Unable to determine	No	Unable to determine
16	Yes	Yes	Yes	Yes	Yes	Yes	Yes	Yes
17	Yes	Yes	Yes	Yes	Yes	No	Yes	Yes
18	Yes	Yes	Unable to determine	Yes	Yes	Yes	Yes	Yes
19	Yes	Yes	Yes	Unable to determine	Unable to determine	Yes	Yes	Yes
20	Yes	Yes	Yes	Yes	Yes	Yes	Yes	Yes
**Internal validity – confounding (selection bias)**								
21	Yes	Yes	Yes	Yes	Yes	Yes	Yes	Yes
22	Yes	Yes	Yes	Yes	Yes	Yes	Yes	Unable to determine
23	Yes	Yes	Yes	Yes	No	No	Yes	Yes
24	Yes	Unable to determine	Yes	Yes	No	No	Yes	Unable to determine
26	No	Yes	Yes	Unable to determine	No	Unable to determine	Yes	Unable to determine
**Power**								
27	No	No	Unable to determine	No	No	No	No	No
Score*	19	23	19	15	14	17	21	13

* Higher score indicates higher quality, with *yes*=1, *no*=0 and *unable to determine*=0
