# Supplementary material for: Use of Technology-Based Tools to Support Adolescents and Young Adults With Chronic Disease: Systematic Review and Meta-Analysis
Source: JMIR Mhealth Uhealth. 2019 Jul 18;7(7):e12042. doi: 10.2196/12042 (PMC6670279; doi:10.2196/12042)
Supplement: Multimedia Appendix 5 [file mhealth_v7i7e12042_app5.pdf]

Multimedia Appendix 5. Characteristics of included interventions.

Study identity	Adolescents and young adults' involvement[a] (intervention duration)	Intervention details
[33]	Yes[b] (6 weeks)	The Web-based self-management program, Challenge your arthritis, was available in Dutch and was based on self-efficacy theory. The program was a password-protected, interactive Web-based self-management program. It included a chat section, home exercises and a discussion board.
[34]	Yes (3 months *as needed*)	The electronic health intervention consisted of a website and a Web-based hospital-based portal. The publicly accessible website was available in Dutch and contained information and tips on 5 themes: (1) treatment and medication; (2) physical exercise, holidays, alcohol, and drugs; (3) relations, sexuality, and pregnancy; (4) dealing with pain, fatigue, and emotions; and (5) study and work. It also contained videos and written stories from other young adults talking about their lives. The hospital portal allowed access to 3 tools: (1) electronic consult with a clinical nurse specialist, (2) personal medical record, and (3) Web-based self-monitoring.
[36]	Yes (—[c])	An option to choose one out of 6 available apps with different functions: (1) record and view data: Diabetes Logger (Simon Fisher) and Diabetes Health Tracker (West Coast Labs); (2) calculate insulin dosage: Insulin Calc (Jagdeep Nagpal) and CPSlider (Dan Pashley); and (3) make notes or set agenda: T1NDA (William Chanter) and You + Your Diabetes (Sarah Youen). Of these, 2 were submitted on iOS, 2 for Android, and 3 were websites.
[37]	Yes, P[d] (20-30 min per week for 8-10 weeks)	The website, Teens Taking Charge: Managing Haemophilia Online, was available in English and French. It was an 8-week program written at a grade 6-7 level. The following topics were addressed: (1) basics of hemophilia, (2) hemophilia management, (3) managing bleeds, (4) complications of hemophilia, (5) mind and body (relaxation, distraction, managing stress, and lifestyle), (6) transition of care, and (7) looking ahead (education and vocation). It was delivered in the form of short videos (3:05-6:07 min each), animations, illustrations, and knowledge quizzes). In addition, the intervention group was contacted by a coach over the telephone every week.
[38]	Yes (—)	The website, SteppingUP, was available in English and contained 9 pages: (1) home page, (2) about transition, (3) clinics and personnel, (4) *top tips*, (5) transition stories (9 video testimonials ranging in length from 2.21-4.48 min), (6) frequently asked questions, (7) essential reading, (8) links, and (9) a photo gallery. The website contained information on various aspects of the transition process and was presented in several formats including short videos and leaflets.
[19]	No (8 months)	The 8-month MD2ME program was based on Bandura's social cognitive theory. It targeted the self-management constructs of monitoring disease symptoms, responding to monitoring with appropriate treatments, and actively working with healthcare providers to manage care. In the first 2 months, theme-based materials outlining common disease management and communication skills and lifestyle tips were delivered via a secure website. Tailored short message service (SMS) messages were delivered via mobile phone to reinforce the Web-delivered intervention. In addition, the intervention group was given access to an automated SMS algorithm to initiate conversation with a health professional to report health concerns.
[41]	No (180 days)	Puff City was a Web-based, tailored asthma management program that provided both normative (*compared with other students*) and ipsative feedback (*compared with your last session*). The program was delivered over 4 consecutive educational computer sessions and focused on 3 core behaviors: (1) controller medication adherence, (2) rescue inhaler availability, and (3) smoking cessation or reduction.
[42]	Yes (—)	The website, Teens Taking Charge: Managing My Transplant Online, contained 3 modules: (1) organ-specific modules (assessment, listed and waiting, what will happen at the time of transplant, preparing for discharge, and self-monitoring and complications), (2) modules with a multiorgan focus (introductions, medications, diet, lifestyle, managing stress, looking forward, and discussion board or ask the experts or interesting facts), and (3) parent modules (impact of transplant and letting go). It was delivered in the form of written materials, peer videos, and animation.
[43]	Yes, P (—)	The congenital heart disease mobile app included user profile, transition checklist component, educational modules, portable medical summary and congenital heart disease diagram, a space for

		medical questions, and a blog space.
[44]	Yes (11 weeks)	YourWay was a Web-based program with 6 multimedia problem-based stories to improve adolescent problem solving, self-management skills, and glycemic control. The story components were designed based on learning, social cognitive, and self-determination theories. Other activities included (1) a personalized homepage, (2) multimedia presentations on the steps of problem solving and how to use the website, (3) social networking via a peer forum, (4) comparison of responses with others, (5) help from a problem-solving expert, and (6) weekly emails that encouraged participation.
[46]	No (4 weeks)	The website, Can-Do-Tude, consisted of diabetes self-management education. The intervention, which was guided by the principles of motivational interviewing, was delivered via a Web-based platform to improve diabetes self-management self-efficacy in adolescents with type 1 diabetes.
[47]	Yes (—)	Kiss myAsthma (The University of Sydney) was a mobile app that focused on asthma management. The features included (1) symptom tracking, (2) mood tracking, (3) recording asthma attacks, and (4) emergency support.
[48]	No (5-6 months)	The internet-based education program was delivered via a website and included an educational module with an asthma-related quiz and an interactive adventure game incorporating numerous virtual asthma-related situations that need to be managed adequately.
[49]	Yes, P (—)	The web-based program, rheumtogrow, contained 5 topic areas: (1) Get Educated: Know the facts about living with Juvenile Arthritis, (2) Take Control: Learn more about healthcare and how to get the support you need, (3) Stay Healthy: Tips on nutrition, exercise, and how to stay healthy, (4) Connect and Share: Hear more from other teens with arthritis and connect with community resources, and (5) My Info.
[50]	— (7-10 days)	A choice from 2 existing mobile apps, AsthmaMD (Mobile Breeze) or AsthmaPulse (Enemra), which were tailored for teens and could be used to track and monitor asthma.
[51]	Yes, P (3 months)	A mobile app that enabled users to track disease patterns, symptoms, triggers, and peak-flow readings and then receive asthma action plan feedback on their current asthma status.
[52]	Yes (—)	Two videos were created: (1) on disclosure to middle school friends and (2) on selecting an appropriate sport based on the severity of hemophilia.
[55]	Yes, P (—)	The Web-based program, Teens Taking Charge: Managing Arthritis Online, was available in English and French. It was a multicomponent, interactive program consisting of education specific to juvenile idiopathic arthritis, self-management strategies, and social support (in the form of a discussion board and video clips) designed for young patients and their parents.
[56]	Yes, P (12 weeks)	The intervention consisted of a website, Teens Taking Charge: Managing Arthritis Online, and regular telephone contact with a trained coach (a non–health care professional with an undergraduate degree in psychology). The Web-based program contained 12 modules: (1) different types of arthritis, (2) understanding how arthritis is diagnosed, (3) arthritis medications, (4) managing symptoms (pain, stiffness, and fatigue), (5) managing stress, (6) managing negative thoughts, (7) relaxation, (8) distraction, (9) other types of care (exercise, nutrition, and splints), (10) self-monitoring and supports, (11) lifestyle issues, and (12) looking ahead (education, vocation, and transitional care issues).
[57]	Yes (—)	iCanCope with Pain program (The Hospital for Sick Children) was integrated across mobile phone and Web-based platforms and consisted of 5 theory-based components,: (1) self-monitoring, (2) smart goal setting, (3) personalized self-management instruction and rehearsal, (4) peer-based social support, and (5) detailed pain education.
[58]	Yes (5 weeks)	The intervention website (TEENCOPE) was an interactive coping skills program and consisted of 5 weekly sessions: (1) self-talk, (2) communication skills, (3) social problem skills, (4) stress management, and (5) conflict. Simultaneously, an educational website (Managing Diabetes) was also designed and consisted of 4 weekly sessions on: (1) glucose control, (2) nutrition, (3) exercise and sick days, and (4) new technology.
[59]	No (4 weeks)	The mobile app, TransitionMate (The Sydney Children's Hospitals Network), was designed to support self-management in young people during their transition from pediatric to adult health care. It consisted of (1) reminder, (2) mood and health tracker, (3) providing visual feedback, (4) help contact information, and (5) memo to record personal notes.

[a]Adolescents and young adults' involvement in the intervention development or refinement process.
[b]Yes: perspectives were sought on the designed intervention.
[c]— indicates not specified or not reported.
[d]Yes, P: perspectives were sought before intervention development.
