# Supplementary material for: Use of Technology-Based Tools to Support Adolescents and Young Adults With Chronic Disease: Systematic Review and Meta-Analysis
Source: JMIR Mhealth Uhealth. 2019 Jul 18;7(7):e12042. doi: 10.2196/12042 (PMC6670279; doi:10.2196/12042)
Supplement: Multimedia Appendix 6 [file mhealth_v7i7e12042_app6.pdf]

Multimedia Appendix 6. A summary of feasibility study or users' perceived acceptance of the intervention (n=16)

Study ID	Feasibility tests / perceived acceptability of the intervention
[33]	The mean feasibility score of the online program was 7.3 (range=5-10) compared to the face-to-face program of 7.7 (range=6-10). Of the 10 participants, 7 rated the program as useful and 9 thought the time invested was worthwhile. All would recommend the program to others, found the program user friendly and liked how the program was presented.
[34]	The mean perceived usefulness score of the website was 7.6 whilst the hospital-based portal was 7.8. Of the 13 participants, 6 intended to use the website again whilst all intended to use the portal again to access their medical records and e-consult.
[36]	Notes/agenda-setting applications were considered more useful (mean=4.10, SD=0.77) for clinic appointments than data logging (mean=3.36, SD=1.08) and insulin dose calculator (mean=3.22, SD=1.28) ($F_{2, 80}$=5.72, *p*=.01). Of the 83 participants, 46 intended to use the application they had chosen, 67 intended to recommend the chosen application to a friend and 65 thought their application was easy to use.
[37]	Of the 11 participants that participated in the satisfaction survey, all participants thought the website was easy to use, 10 would recommend it to others and 9 liked the content of the intervention. In relation to telephone coaching, 9 participants felt the frequency of contact was appropriate.
[42]	Participants felt that the content of the Web site was easy to read and understand, it was detailed and contained relevant information. They liked the videos of the other patients talking about their experiences but wanted more pictures and graphics, a discussion forum, games, and music added to the website. They would revisit the website and would recommend it to other patients.
[44]	The stories were rated as highly relevant (mean=4.4, SD=0.5). Of the 35 participants that were reached for a post-completion interview, 63% gave the intervention an overall grade of "A" and the remainder gave it a "B".
[46]	Intervention satisfaction survey was conducted with 3 participants who completed the intervention. They found the intervention to be helpful in managing diabetes, enjoyable, interesting and easy to use. They thought the time spent doing the intervention was worthwhile and were likely manage their disease differently after completing the intervention.
[47]	Of the nine participants that participated in the user feedback workshop, all reported that they would recommend the application to others. They liked the mental health support features in the application.
[50]	Participants appreciated visual aids provided by the applications, especially measuring their peak expiratory flow and putting these data into the application, which resulted in a color-coded zone for their symptoms. Participants valued the option to share asthma reports with selected e-mail recipients although they were unsure if their reports were emailed successfully. Additionally, they described the application interfaces as 'boring, too serious, and unappealing'. The colors, graphics and font used in the applications were unsatisfactory.
[51]	After using the mobile application, 90% of participants reported that their ability to manage their asthma improved, 90% liked viewing their asthma patterns over time, 85% noted that the application prompted them to be more adherent to their medications, 65% valued the option of revisiting or sharing their notes with the physician to explain asthma patterns and 40% appreciated the specific instructions for emergency medication.
[52]	Most of the participants (number not specified in the report) reported that they easily understood the main message of the videos, agreed that both videos would have been helpful to them when they were younger and would have encouraged them to seek more information.
[55]	Of the 19 adolescent participants, 89% felt that the content was relevant and 74% rated the content as trustworthy. All participants felt that the adaptive and interactive features of the website would enhance engagement with the program. All participants commented that online discussion board within the site would help ease the feelings of hopelessness and isolation. 84% would use the website in the future. Most participants felt the program would have been useful

	when they were first diagnosed.
[56]	The 20 participants in the intervention group rated the website as easy to use and highly acceptable. Most participants felt the e-mail system with the coach and technical assistance was helpful. They would recommend the website to other patients and their families.
[57]	Of the 7 adolescent participants, 6 endorsed the proposed iCanCope with Pain™ architecture as acceptable for meeting their pain self-management needs.
[58]	Ten participants were involved in the last phase of evaluations of both Managing Diabetes and TEENCOPE sites. The response was positive for both websites, with 90% of the participants indicating that they liked the websites, 90% thought both were clear and interesting and 80% thought the sites were appropriate for teenagers.
[59]	The application was evaluated by 9 participants and achieved an average overall score of 7.4 out of 10. The design of the application was scored 3.4 out of 5 whilst the ease of use obtained a score of 8.1 out of 10.
