# Supplementary material for: Use of Technology-Based Tools to Support Adolescents and Young Adults With Chronic Disease: Systematic Review and Meta-Analysis
Source: JMIR Mhealth Uhealth. 2019 Jul 18;7(7):e12042. doi: 10.2196/12042 (PMC6670279; doi:10.2196/12042)
Supplement: Multimedia Appendix 7 [file mhealth_v7i7e12042_app7.pdf]

Multimedia Appendix 7. A summary of intervention efficacy evaluations (n = 8)

Study ID	Statistical evaluations of intervention efficacy
[37]	Between groups: Compared to the control group, hemophilia-specific knowledge gain increased significantly in the intervention group across the study period (*P*=.01). There were no group differences found in relation to health-related quality of life, self-efficacy or self-management preparedness of transition.<br>Within group: Within the intervention group, there was a significant increment in the pre- and postscores of hemophilia-specific knowledge (*P*=.004), average self-efficacy (*P* = 007) and self-management preparedness of transition (*P*=.02).
[19]	Between groups: Compared to the control group, there were significant improvements in the intervention group across the study period in disease self-management (*P*=.02), health-related self-efficacy (*P=.02*) and patient-initiated communications with the healthcare team (*P*<.001). No significant changes were found in disease status, functional performance, and quality of life over the study period by treatment group.
[41]	Cross-sectional between-group evaluations at 12 months: Compared to the control group, significantly less negative change was observed for rescue inhaler availability (pairwise comparison *P*=.003) in the intervention group. After adjusting for baseline measures, baseline asthma severity, school of enrollment, and contact with the referral coordinator, the intervention group had significantly fewer symptom days (*P*=.003), symptom nights (*P*=.009), school days missed (*P*=.006), days of restricted activity (*P*=.02) and asthma hospitalizations (*P*=.01) than the control group. No group differences were found in the score of controller medication adherence, smoking behaviors, days of changed plans, asthma-related visits to the emergency department and quality of life.
[44]	Between groups: Intention-to-treat analyses revealed no statistically significant group differences in change over time. However, as-treated analysis revealed that the intervention group scored better than the control group at problem solving (d=.30, 95% CI 0.14 to 0.49; *P*=.23) and self-management skills (d=.64, 95% CI 0.53–0.79; *P*=.02) in addition to having a lower blood glucose level (d=-.28, 95% CI 0.25–0.67; P=.27).
[46]	One group pre-posttest: Participants (n=3) demonstrated overall improvements in diabetes self-management self-efficacy (Z=-2.952, *P*=.007).
[48]	Between groups: Morbidity cost savings in the SPMP (control) group were lower when compared to IEP+SPMP (intervention) group. Adding the IEP to the SPMP yielded incremental morbidity cost savings of 160€ at an average cost of 44€ when compared to the SPMP alone, which translates into a benefit-cost ratio of 3.65. At 6 months, the number of emergency department visits was significantly lower in the IEP+SPMP group than the SPMP group (*P*=.03) whilst other outcomes did not differ between groups: number of physician consultations, hospital days, caregivers' working days lost, therapy costs, daily use of rescue medication and school absenteeism. No group differences were observed at 12 months in all outcomes.<br>Within group: When compared to baseline data, the IEP+SPMP group had significant reductions in asthma-related emergency treatments (*P*=.02) at 6 months and daily use of rescue medication (*P*=.02) at 12 months. Both IEP+SPMP and SPMP groups had significantly reduced number of physician consultations and school absenteeism at 6 and 12 months compared to baseline. Number of emergencies and daily cost of therapy were significantly reduced at 12 months when compared to baseline. Quality of life increased significantly (*P*<.05) in both IEP+SPMP and SPMP groups across the study period.
[56]	Between groups: Across the study period, the intervention group had significantly higher knowledge score (*P*<.001, d=1.32) and lower average weekly pain intensity (*P*=.03, d=.78) than the control group. There were no significant group differences in quality of life, treatment adherence, self-efficacy and perceived stress level.
[58]	Between groups: No group differences were found in diabetes self-efficacy, coping skills, quality of life, perceived stress and communication skills across the study period.
